# Supplementary material for: Y-Box-Binding Proteins Have a Dual Impact on Cellular Translation
Source: Int J Mol Sci. 2024 Feb 1;25(3):1736. doi: 10.3390/ijms25031736 (PMC10855678; doi:10.3390/ijms25031736)
Supplement: Supplementary file 1 [file ijms-25-01736-s001.zip › ijms-2806949-supplementary.pdf]

Supplementary information. Contents.

1. Supplementary Methods
   1.1. Measuring the total translation level.
   1.2. Quantitative reverse transcription PCR (qRT-PCR).

2. Figures and Tables
   2.1. **Table S1.** List of snoRNAs with significant changes of Ribo counts.
   2.2. **Table S2.** Description of samples.
   2.3. **Table S3.** Primers for qRT-PCR.
   2.4. **Figure S1.** The total translation level measured by AHA incorporation.
   2.5. **Figure S2.** Changes of translation-related genes.
   2.6. **Figure S3.** PCA and metagene profiles around start codons.
   2.7. **Figure S4.** Verification of RNA-Seq data.
   2.8. **Figure S5.** Verification of Ribo-Seq data.
   2.9. **Table S4.** Summary of Ribo-Seq data verification.

1.1. Measuring the total translation level (for Figure S1)

The total translation level was determined using the incorporation of azidohomoalanine (AHA) into newly synthesized proteins, followed by its crosslinking with an DBCO (dibenzocyclooctyne) derivative of the fluorescent dye Alexa Fluor 488. The cells were cultured on a 6-well plate to a density of 60-70%. Then DMEM was replaced by methionine-free DMEM, and the cells were incubated in the absence of FBS for 45 min under standard conditions. Then the reaction mixture was replaced by fresh methionine-free DMEM containing azidohomoalanine (50 μM). After 3 h incubation, the cells were washed with ice-cold PBS, and 200 μl of lysis buffer (1% SDS in 50 mM Tris-HCl (pH 8.0) and Benzonase (250 U/ml)) was added to each well of the plate. A 200 μg sample of lysate was diluted with PBS to a volume of 100 μl, mixed with 100 μl of PBS buffer containing 40 μM DBCO-Alexa488 (Jena Bioscience), and incubated at room temperature for 1 h in the dark. After incubation, the reaction mixture was supplemented with 600 μl methanol, 150 μl chloroform, and 400 μl water, well-mixed and subjected to centrifugation at 13,000 g for 5 min. Then the aqueous phase was removed and replaced by 450 μl methanol, and centrifugation at 13,000 g was continued for 5 min. The methanol washing was repeated twice, after which the pellet was let dry for 1 h and then dissolved in 200 μl of 1.5x sample buffer for SDS-PAGE.

For all samples, equal total protein amounts were separated by SDS-PAGE and Alexa Fluor 488-labeled proteins were detected on a ChemiDoc MP Imaging System (Bio-Rad) and processed using the OptiQuant Software (Packard Instruments).

1.2. Quantitative reverse transcription PCR (qRT-PCR) (for Figures S4 and S5)

Total RNA was obtained from cells or sucrose gradient fractions using TRIzol LS Reagent (Thermo Fisher Scientific) according to the manufacturer's recommendations. Up to 1 μg of total RNA was used in reverse transcription reaction with Maxima H Minus Reverse Transcriptase (Thermo Fisher Scientific). Quantitative real-time PCR (qRT-PCR) was performed on a DTlite Real-Time PCR System (DNA Technology) using qPCRmix-HS SYBR+LowROX reaction mixture (Evrogen). A 25 μl aliquot of final reaction mixture contained 1/40 of the RT reaction mixture and 0.4 μM primers (see Table S3). The following cycling conditions were used: 5 min at 95°C followed by 50 cycles at 95°C for 10 sec, 59°C for 20 sec, and 72°C for 10 sec. For the total RNA samples, the statistical data analysis and assessment of differences in gene expression were performed using the REST2009 software; the reference genes were *BTF3* and *ALDH16A*. For sucrose gradient fractions, transcript abundance values were normalized to those of *Nluc* mRNA.

Ensemble Gene ID	gene_name	gene_type	YB-1 vs ΔΔ Ribo_log2FC	YB-3 vs ΔΔ Ribo_log2FC	YB-1 vs ΔΔ RNA_log2FC	YB-3 vs ΔΔ RNA_log2FC
ENSG00000238795	SCARNA12	snoRNA	2.58	3.01	-0.12	-0.08
ENSG00000220988	SNORD88C	snoRNA	2.34	2.93	-0.46	-0.24
ENSG00000207523	SNORA66	snoRNA	2.33	2.38	0.35	-0.13
ENSG00000275996	SNORD27	snoRNA	1.97	2.22	-0.09	0.32
ENSG00000200084	SNORD68	snoRNA	1.79	1.39	-0.35	-0.26
ENSG00000206680	SNORD21	snoRNA	1.74	1.67	-0.32	-0.34
ENSG00000207405	SNORA64	snoRNA	1.62	1.92	-0.02	0.31
ENSG00000208892	SNORA49	snoRNA	1.55	2.08	-0.12	0.44
ENSG00000201302	SNORA65	snoRNA	1.47	2.12	-0.20	0.42
ENSG00000200354	SNORA71D	snoRNA	1.22	1.73	0.38	0.23
ENSG00000277512	SNORD65	snoRNA	1.15	0.91	-0.23	0.08
ENSG00000209480	SNORD83B	snoRNA	1.09	1.08	-0.46	-0.06
ENSG00000276788	SNORD26	snoRNA	1.09	1.15	-0.54	0.19
ENSG00000212195	RF00012	snoRNA	0.96	1.25	0.27	-0.24
ENSG00000201754	SNORD52	snoRNA	0.72	0.91	0.27	-0.21
ENSG00000225091	SNORA71A	snoRNA	0.55	-1.21	-0.09	0.04
ENSG00000235408	SNORA71B	snoRNA	0.47	0.53	0.03	0.06
ENSG00000200087	SNORA73B	snoRNA	0.45	1.68	-0.11	0.41
ENSG00000201998	SNORA23	snoRNA	-1.21	-0.62	0.01	0.20
ENSG00000238835	SCARNA18	snoRNA	-1.37	-1.70	-0.14	0.26

Table S1. List of snoRNAs with significant changes of Ribo counts. Log2 Fold changes of Ribo-counts and RNA counts in YB-1/YB-3-expressing cells compared with ΔΔ cells are presented.

Sample	Library type	Cell line	Genotype	Sequencing type	Instrument model	Sequencing batch	Library preparation batch	Total gene-level counts*
HEK293T ΔYB-1ΔYB-3, Ribo-Seq, rep1	Ribo-Seq	HEK293T	YBX1 knockout & YBX3 knockout	single-end	Illumina HiSeq 2000	batch1, batch2: HiSeq 2000	batch 1	8 981 414
HEK293T ΔYB-1ΔYB-3, Ribo-Seq, rep2	Ribo-Seq	HEK293T	YBX1 knockout & YBX3 knockout	single-end	Illumina NextSeq 500, HiSeq 2000	batch1: NextSeq 500, batch2: HiSeq 2000	batch 2	6 983 165
HEK293T ΔYB-1ΔYB-3, Ribo-Seq, rep3	Ribo-Seq	HEK293T	YBX1 knockout & YBX3 knockout	single-end	Illumina HiSeq 2000	batch1, batch2: HiSeq 2000	batch 1	8 375 210
HEK293T ΔYB-1ΔYB-3, RNA-Seq, rep1	RNA-Seq	HEK293T	YBX1 knockout & YBX3 knockout	single-end	Illumina NextSeq 500, HiSeq 2000	batch1: NextSeq 500, batch2: HiSeq 2000	batch 2	9 613 330
HEK293T ΔYB-1ΔYB-3, RNA-Seq, rep2	RNA-Seq	HEK293T	YBX1 knockout & YBX3 knockout	single-end	Illumina NextSeq 500, HiSeq 2000	batch1: NextSeq 500, batch2: HiSeq 2000	batch 2	7 908 646
HEK293T ΔYB-1ΔYB-3, RNA-Seq, rep3	RNA-Seq	HEK293T	YBX1 knockout & YBX3 knockout	single-end	Illumina HiSeq 2000	batch1, batch2: HiSeq 2000	batch 1	5 861 021
HEK293T ΔYB-1ΔYB-3 + YB-1, Ribo-Seq, rep1	Ribo-Seq	HEK293T	YBX1 complementation in YBX1 knockout & YBX3 knockout	single-end	Illumina NextSeq 500, HiSeq 2000	batch1: NextSeq 500, batch2: HiSeq 2000	batch 2	4 056 517
HEK293T ΔYB-1ΔYB-3 + YB-1, Ribo-Seq, rep2	Ribo-Seq	HEK293T	YBX1 complementation in YBX1 knockout & YBX3 knockout	single-end	Illumina HiSeq 2000	batch1, batch2: HiSeq 2000	batch 1	9 514 818
HEK293T ΔYB-1ΔYB-3 + YB-1, RNA-Seq, rep1	RNA-Seq	HEK293T	YBX1 complementation in YBX1 knockout & YBX3 knockout	single-end	Illumina NextSeq 500, HiSeq 2000	batch1: NextSeq 500, batch2: HiSeq 2000	batch 2	8 748 187
HEK293T ΔYB-1ΔYB-3 + YB-1, RNA-Seq, rep2	RNA-Seq	HEK293T	YBX1 complementation in YBX1 knockout & YBX3 knockout	single-end	Illumina HiSeq 2000	batch1: HiSeq 2000	batch 1	6 594 585
HEK293T ΔYB-1ΔYB-3 + YB-1 + YB-3, Ribo-Seq, rep1	Ribo-Seq	HEK293T	YBX1 & YBX3 complementation in YBX1 knockout & YBX3 knockout	single-end	Illumina NextSeq 500, HiSeq 2000	batch1: NextSeq 500, batch2: HiSeq 2000	batch 2	8 720 180

HEK293T ΔYB-1ΔYB-3 + YB-1 + YB-3, Ribo-Seq, rep2	Ribo-Seq	HEK293T	YBX1 & YBX3 complementation in YBX1 knockout & YBX3 knockout	single-end	Illumina HiSeq 2000	batch1, batch2: HiSeq 2000	batch 1	7 692 095
HEK293T ΔYB-1ΔYB-3 + YB-1 + YB-3, Ribo-Seq, rep3	Ribo-Seq	HEK293T	YBX1 & YBX3 complementation in YBX1 knockout & YBX3 knockout	single-end	Illumina HiSeq 2000	batch1, batch2: HiSeq 2000	batch 1	7 597 906
HEK293T ΔYB-1ΔYB-3 + YB-1 + YB-3, RNA-Seq, rep1	RNA-Seq	HEK293T	YBX1 & YBX3 complementation in YBX1 knockout & YBX3 knockout	single-end	Illumina NextSeq 500, HiSeq 2000	batch1: NextSeq 500, batch2: HiSeq 2000	batch 2	6 118 372
HEK293T ΔYB-1ΔYB-3 + YB-1 + YB-3, RNA-Seq, rep2	RNA-Seq	HEK293T	YBX1 & YBX3 complementation in YBX1 knockout & YBX3 knockout	single-end	Illumina HiSeq 2000	batch1: HiSeq 2000	batch 1	7 499 764
HEK293T ΔYB-1ΔYB-3 + YB-3, Ribo-Seq, rep1	Ribo-Seq	HEK293T	YBX3 complementation in YBX1 knockout & YBX3 knockout	single-end	Illumina NextSeq 500, HiSeq 2000	batch1: NextSeq 500, batch2: HiSeq 2000	batch 2	6 404 301
HEK293T ΔYB-1ΔYB-3 + YB-3, Ribo-Seq, rep2	Ribo-Seq	HEK293T	YBX3 complementation in YBX1 knockout & YBX3 knockout	single-end	Illumina HiSeq 2000	batch1, batch2: HiSeq 2000	batch 1	6 434 555
HEK293T ΔYB-1ΔYB-3 + YB-3, Ribo-Seq, rep3	Ribo-Seq	HEK293T	YBX3 complementation in YBX1 knockout & YBX3 knockout	single-end	Illumina HiSeq 2000	batch1, batch2: HiSeq 2000	batch 1	7 607 432
HEK293T ΔYB-1ΔYB-3 + YB-3, RNA-Seq, rep1	RNA-Seq	HEK293T	YBX3 complementation in YBX1 knockout & YBX3 knockout	single-end	Illumina NextSeq 500, HiSeq 2000	batch1: NextSeq 500, batch2: HiSeq 2000	batch 2	7 424 473
HEK293T ΔYB-1ΔYB-3 + YB-3, RNA-Seq, rep2	RNA-Seq	HEK293T	YBX3 complementation in YBX1 knockout & YBX3 knockout	single-end	Illumina HiSeq 2000	batch1: HiSeq 2000	batch 1	7 889 558
HEK293T, YB-1 PAR-CLIP, repA	PAR-CLIP	HEK293T	wild type	single-end	Illumina HiSeq 2000	batch1: HiSeq 2000	batch 3	464 510
HEK293T, YB-1 PAR-CLIP, repB	PAR-CLIP	HEK293T	wild type	single-end	Illumina HiSeq 2000	batch1: HiSeq 2000	batch 3	1 223 792

Table S2. Description of samples. *The reads statistics show the total gene-level read counts for RNA-Seq and Ribo-Seq samples, and total mapped reads for PAR-CLIP samples.

Gene name	Primer sequence	
ADGRB1	For_ADGRB1	ACCTGTTGGCAGAGGAGAATCG
	Rev_ADGRB1	GGTTGTCTGTCACCTGGTATGC
AGO4	For_AGO4	ACAAGGTGCAGACAGTGTGGAG
	Rev_AGO4	TCTCCAACACGTTTCACCTCCG
ALDH16A1	For_ALDH16A1	GCCATGTGGTATTTCGGATCA
	Rev_ALDH16A1	TGAGGTGTCCAAAATGCAGTAG
ANKRD1	For_ANKRD1	CGACTCCTGATTATGTATGGCGC
	Rev_ANKRD1	GCTTTGGTTCCATTCTGCCAGTG
BCL2	For_BCL2	ATCGCCCTGTGGATGACTGAGT
	Rev_BCL2	GCCAGGAGAAATCAAACAGAGGC
BCLAF1	For_BCLAF1	CCTAAACGAGCGGTTCACTTCG
	Rev_BCLAF1	GCTAAACGGGTATGCTTCCTCAG
BTF3	For_BTF3	CAGAAAGAAGAAGGTGGTTCATAGA
	Rev_BTF3	ATGCCTGAACTTTAGGGTTGTT
CCNA2	For_CCNA2	TGGATGGTAGTTTTGAGTCACCA
	Rev_CCNA2	ATTTAACCTCCATTTCCCTAAGGTATGT
CCND1	For_CCND1	GTGAACAAGCTCAAGTGGAACCT
	Rev_CCND1	CATGGAGGGCGGATTGGAAATG
CDK4	For_CDK4	TGGGCAAAATCTTTGACCTGATTGG
	Rev_CDK4	AAAGGCAGAGATTCGCTTGTGTG
CDK6	For_CDK6	GATGGCTCTAACCTCAGTGGTC
	Rev_CDK6	AACTTCCACGAAAAAGAGGCTTTC
CDKN1A	For_CDKN1A	AGGTGGACCTGGAGACTCTCAG
	Rev_CDKN1A	TCCTCTTGGAGAAGATCAGCCG
COLEC12	For_COLEC12	AGACTCCAAGCATGGTCAGCTC
	Rev_COLEC12	CCTTTCTGTCCCTTGTTGCCAG
CRABP2	For_CRABP2	TTGAGGAGCAGACTGTGGATGG
	Rev_CRABP2	GTTCTCTGGTCCACGAGGTCTT
DDR2	For_DDR2	AACGAGAGTGCCACCAATGGCT
	Rev_DDR2	ACTCACTGGCTTCAGAGCGGAA
E2F2	For_E2F2	TTCATTTACCTCCTGAGCGAGT
	Rev_E2F2	TCTTCAAACATTCCCCTGCCTAC
EEF2	For_EEF2	AAGCAGTTTGCCGAGATGTATG
	Rev_EEF2	GCTGGGTCAAAGTACCTGTCA
eIF1	For_eIF1	GTGAAGGCGTTTAAGAAAAAGTTTG
	Rev_eIF1	GGTCACCCTGTAGCTGAATTAC
EIF4EBP2	For_EIF4EBP2	AGTCACTAGCCCTGGCACCTTA
	Rev_EIF4EBP2	CTTGCAGGAGAGTCAGATGTCC
EIF5B	For_EIF5B	AACGGAGGATTGAGAAACGGCG
	Rev_EIF5B	TTCCCTGTGTCCACATGCCCAA
EVL	For_EVL	GGACCTCAAAGTCCGATGCCAA
	Rev_EVL	TGTCTGACTGGGAGGCTGCTTT
FLT1	For_FLT1	CCTGCAAGATTCAGGCACCTATG
	Rev_FLT1	GTTTCGCAGGAGGTATGGTGCT
FOXF2	For_FOXF2	CACTCCAGCATGTCCTCCTACT
	Rev_FOXF2	CACTGGAGTAGAGTGATGCTGG
FTL	For_FTL	TACGAGCGTCTCCTGAAGATGC
	Rev_FTL	GGTTCAGCTTTTTCTCCAGGGC

GARS	For_GARS	TCCATGTACGAGAAGGAGATGA
	Rev_GARS	GTCAGGGCTTCCGATAATTCC
HOXB2	For_HOXB2	GAAATCCGCCAAGAAACCCAGC
	Rev_HOXB2	CTGCGTGTTGGTGTAAGCCGTG
JUNB	For_JUNB	CGATCTGCACAAGATGAACCACG
	Rev_JUNB	CTGCTGAGGTTGGTGTAAACGG
LGR5	For_LGR5	CCTGCTTGACTTTGAGGAAGACC
	Rev_LGR5	CCAGCCATCAAGCAGGTGTTCA
LYSMD4	For_LYSMD4	GACCTAATGGGGCTGGAGATAC
	Rev_LYSMD4	GTCTTGTTCTCTGATGAAGTTGTTG
MOXD1	For_MOXD1	GCACTTTGGAGTGCCTGGAAGA
	Rev_MOXD1	AATGACGCAGCCTGATGCCTCT
MT-CYB	For_MT-CYB	GTGATCTGAGTTCAGACCGGAGTA
	Rev_MT-CYB	ATTTACGGGGGAAGGCGCT
NDUFA1	For_NDUFA1	GTACATCCACAGGTTCACTAACG
	Rev_NDUFA1	TTCTCCAAACCCTTTGACACATAG
Nluc	For_Nluc	GCTGTTCCGAGTAACCATCAAC
	Rev_NLuc	GGTCCATACCGCTTTCTTGTG
OBSCN	For_OBSCN	CAGCTCCATTGTCAGGGTGCAT
	Rev_OBSCN	GGACGTTGTTTCCATAGCACCAC
PABPC1	For_PABP	CCAACCCTGTAATCAACCCCTA
	Rev_PABP	TGGAACCTGTGAAGAAGCTGGT
RPS2	For_RPS2	CGATGACTGCTACACCTCAGCC
	Rev_ RPS2	CTCCTGATAGGGAGACTTGGTG
SPON1	For_SPON1	TGTAGCTGACCTGGCTCCAGAA
	Rev_SPON1	CGCATCCTCTTGCCTTTGTCAC
TOMM7	For_TOMM7	TTGCTGTAAGGGGTCCTCCC
	Rev_TOMM7	GCAAACTGGCTCCCCTTGAA

Table S3. Primers for qRT-PCR.

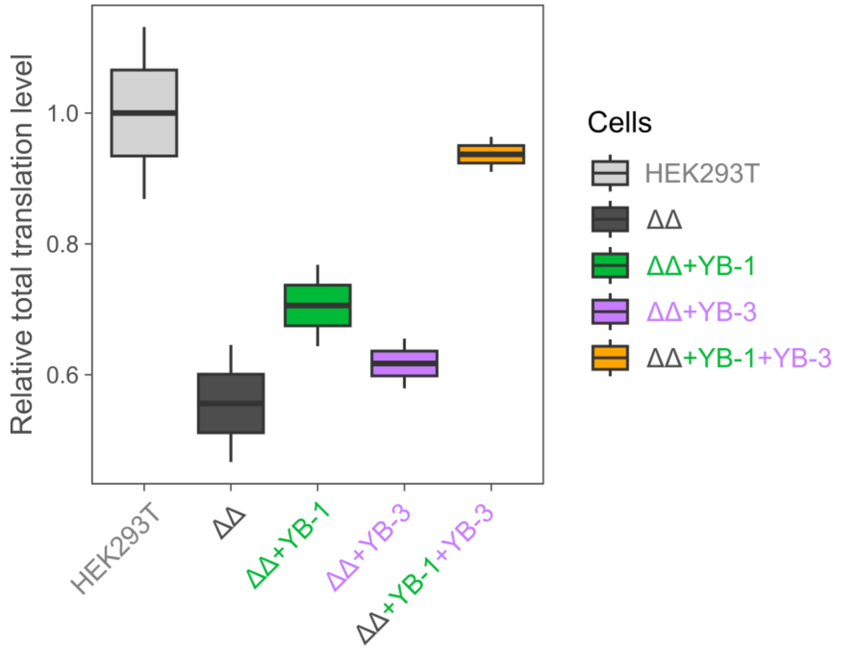

Figure S1. The total translation level was determined by the incorporation of azidohomoalanine (AHA) into newly synthesized proteins, followed by its crosslinking with a DBCO (dibenzocyclooctyne) derivative of the fluorescent dye Alexa Fluor 488 (see Supplementary Methods 1.1). The values were computed as follows: for each biological replicate, the values were normalized to the mean value of a particular replicate; the obtained values were then normalized across experiments to the mean value of HEK293T cells.

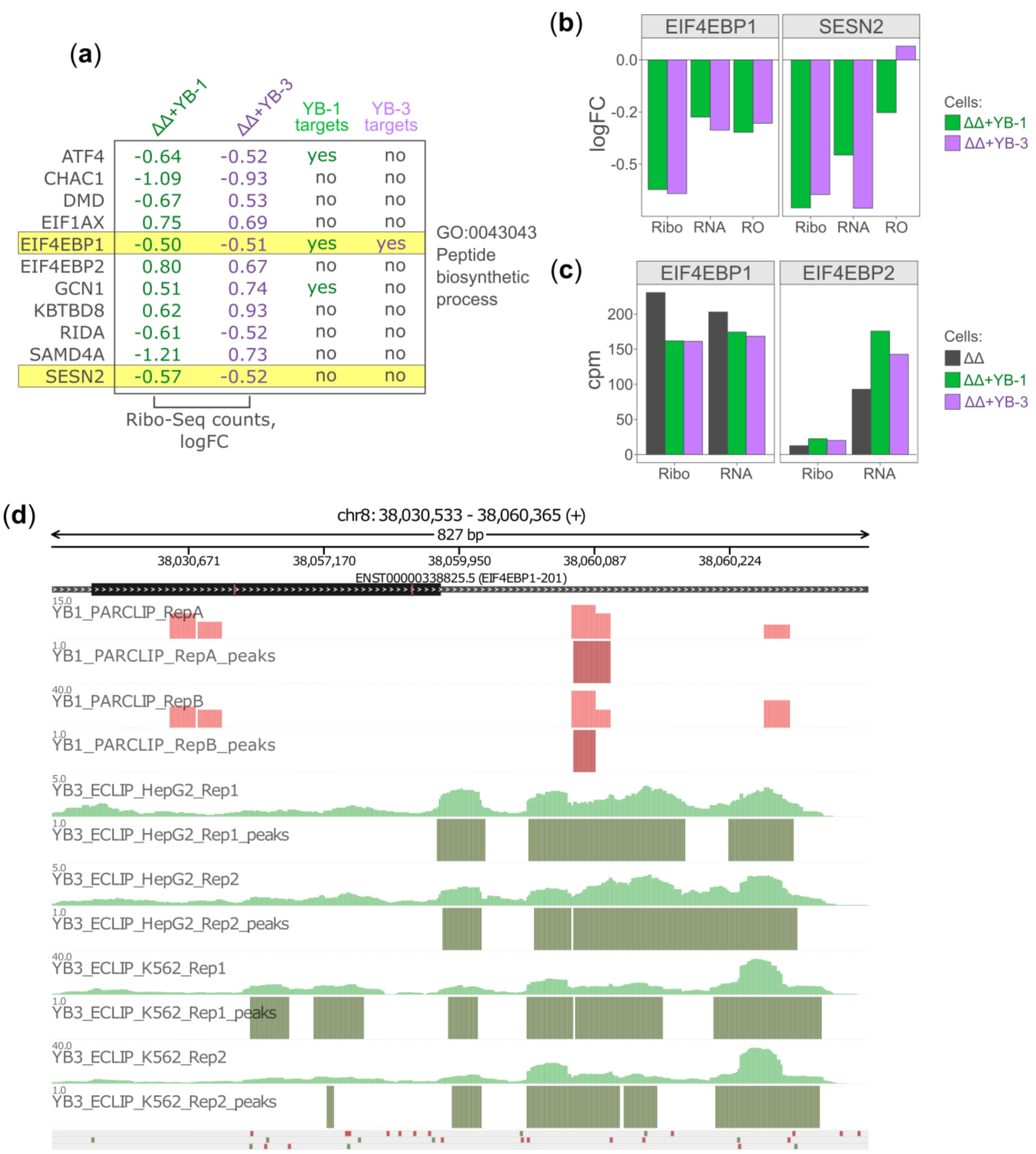

Figure S2. Changes of translation-related genes. (a), List of translation-related genes (extracted from GO: 0043043) with significant changes of Ribo counts (FDR < 0.05, 0.5 < log2FC< -0.5). Log2 Fold changes of Ribo-counts in YB-1/YB-3-expressing cells compared with ΔΔ cells are presented. The YB-1- or YB-3-bound mRNAs (mRNA targets) identified by PAR-CLIP or eCLIP, respectively. **(b),** The changes of ribosome footprint counts (Ribo), RNA abundance (RNA), and ribosome occupancy (RO) in YB-1- and YB-3-expressing cells for EIF4EBP1 and SESN2 are presented. **(c),** The normalized counts (cpm, count per million) of Ribo-Seq (Ribo) and RNA-Seq (RNA) for EIF4EBP1 and EIF4EBP2 are presented. **(d),** The YB-1 PAR-CLIP and YB-3 eCLIP coverage and peaks positions for the EIF4EBP1 gene are visualized using svis4get software [doi: 10.1186/s12859-019-2706-8].

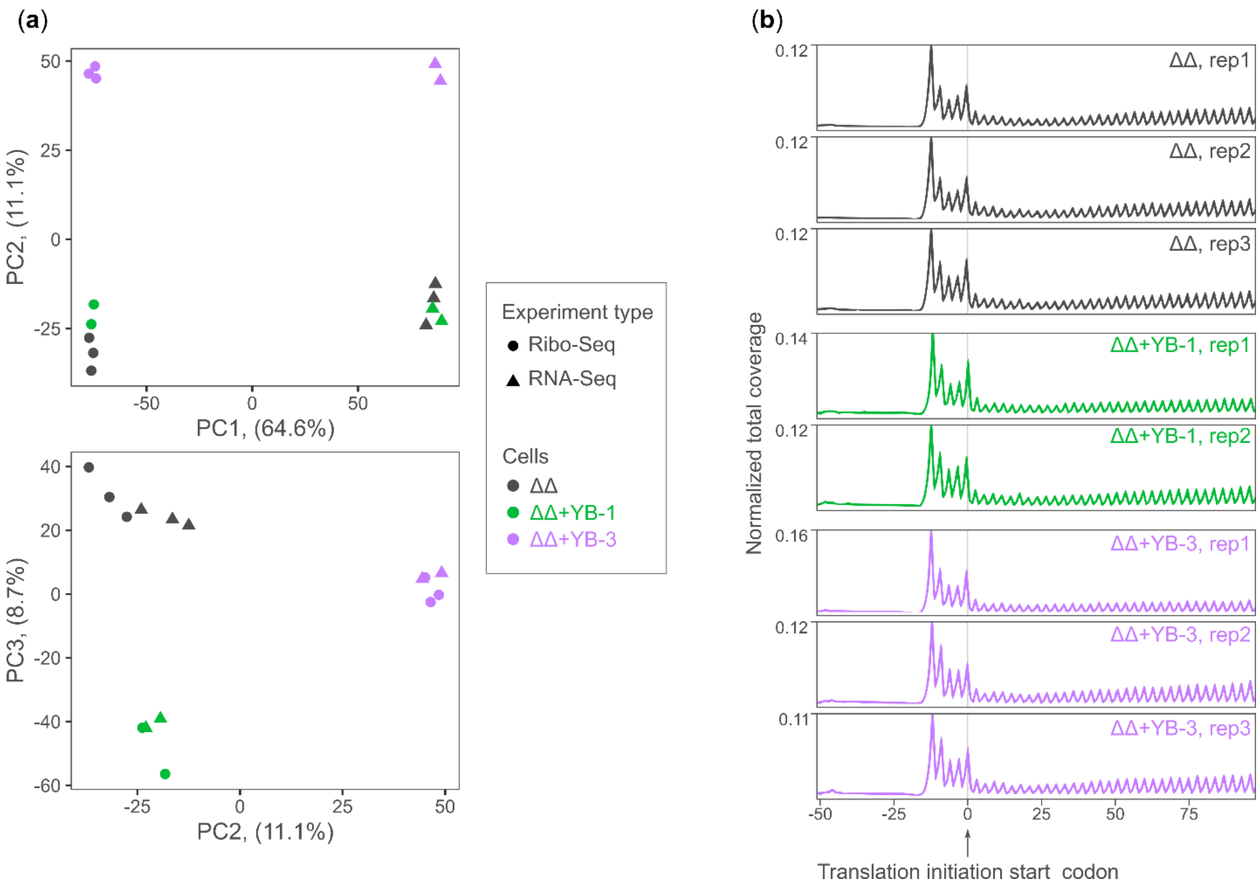

Figure S3. PCA and metagene profiles around start codons. (a), Principal component analysis (PCA) of normalized RNA-Seq and Ribo-Seq data after batch-correction. The percentage of variation explained by a particular principal component (PC) is indicated in the axis label. The point shape and coloring are consistent with the experiment and cell type. **(b),** Metagene profiles representing 5' ends of ribosome footprints are summed up across expressed annotated coding transcripts and anchored at the translation initiation sites. Each profile is normalized to total counts within a 150 nt [-50;+100] window surrounding start codons. The plots demonstrate clear 3-base periodicity within the CDS regions.

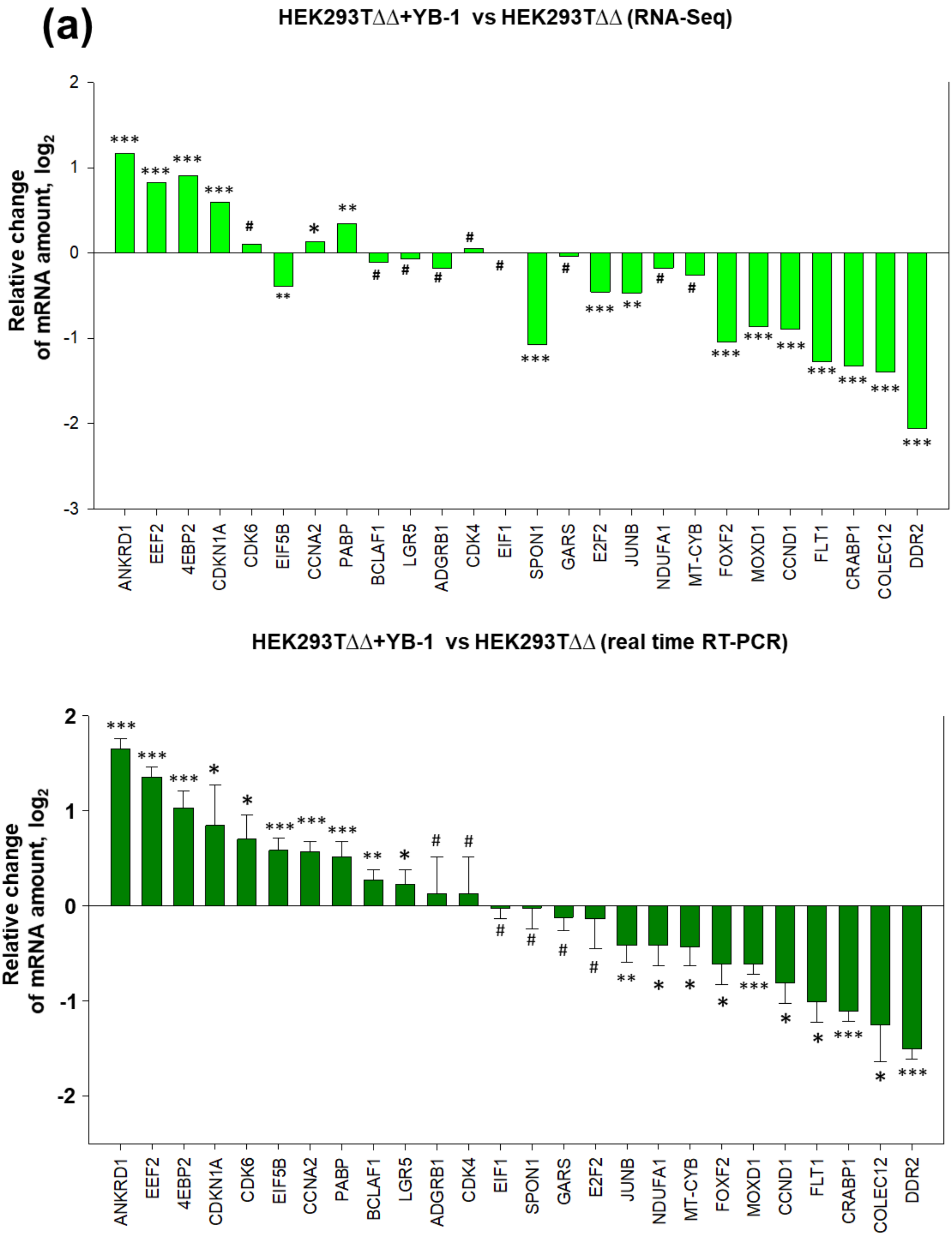

Figure S4. Verification of RNA-Seq data by real-time RT-PCR. (a), HEK293TΔΔ+YB-1 versus HEK293TΔΔ; **(b, next page)** HEK293TΔΔ+YB-3 versus HEK293TΔΔ. Top panel – RNA-Seq data (average of two biological replicates). Bottom panel – real-time RT-PCR results (5 replicates, errors – standard deviation, * - $p < 0.05$, ** - $p < 0.01$, *** - $p < 0.001$, # - non-significant).

(b)

HEK293TΔΔ+YB-3 vs HEK293TΔΔ (RNA-Seq)

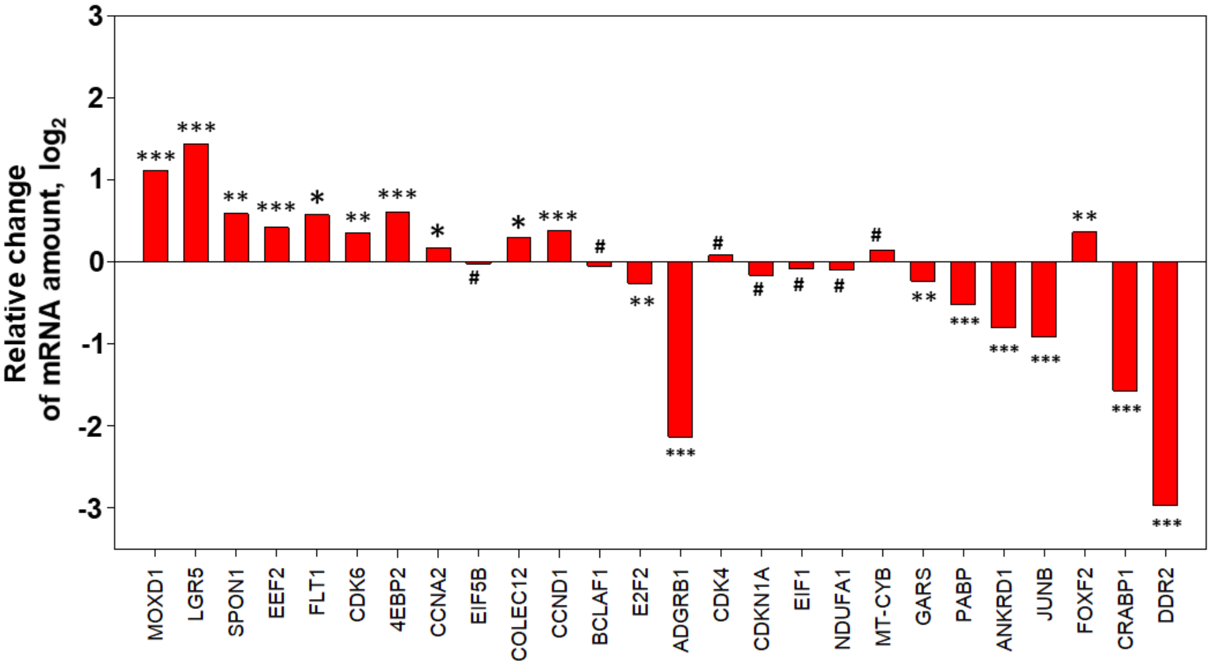

HEK293TΔΔ+YB-3 vs HEK293TΔΔ (real time RT-PCR)

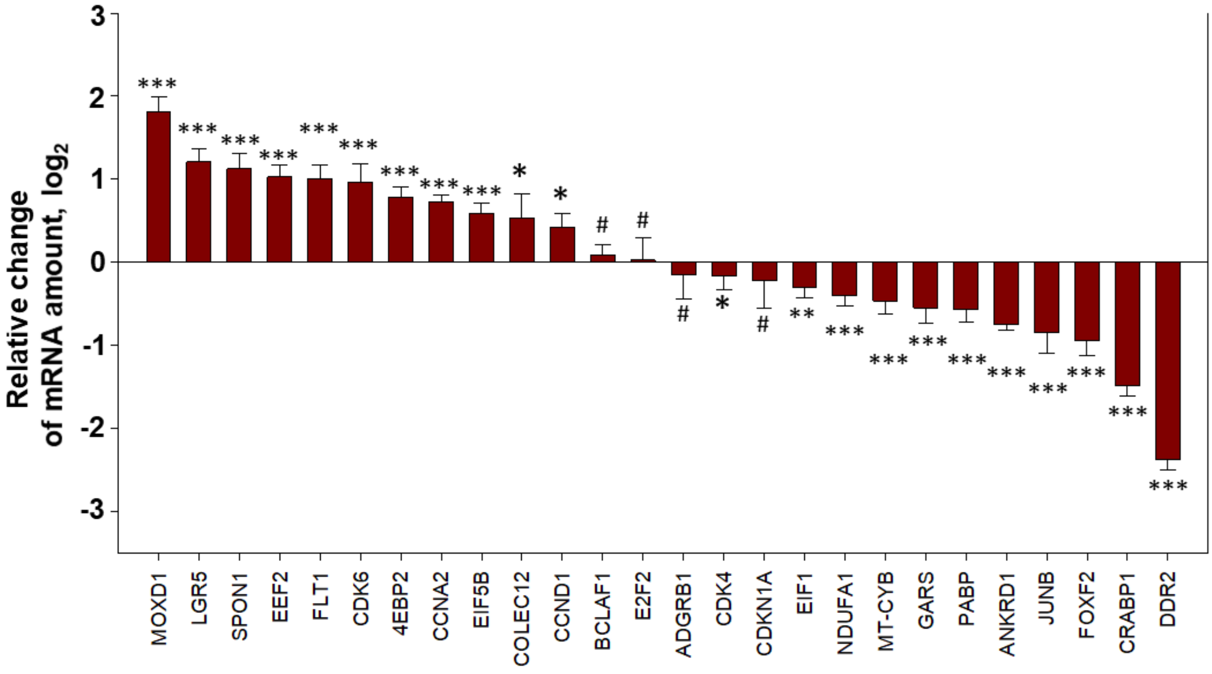

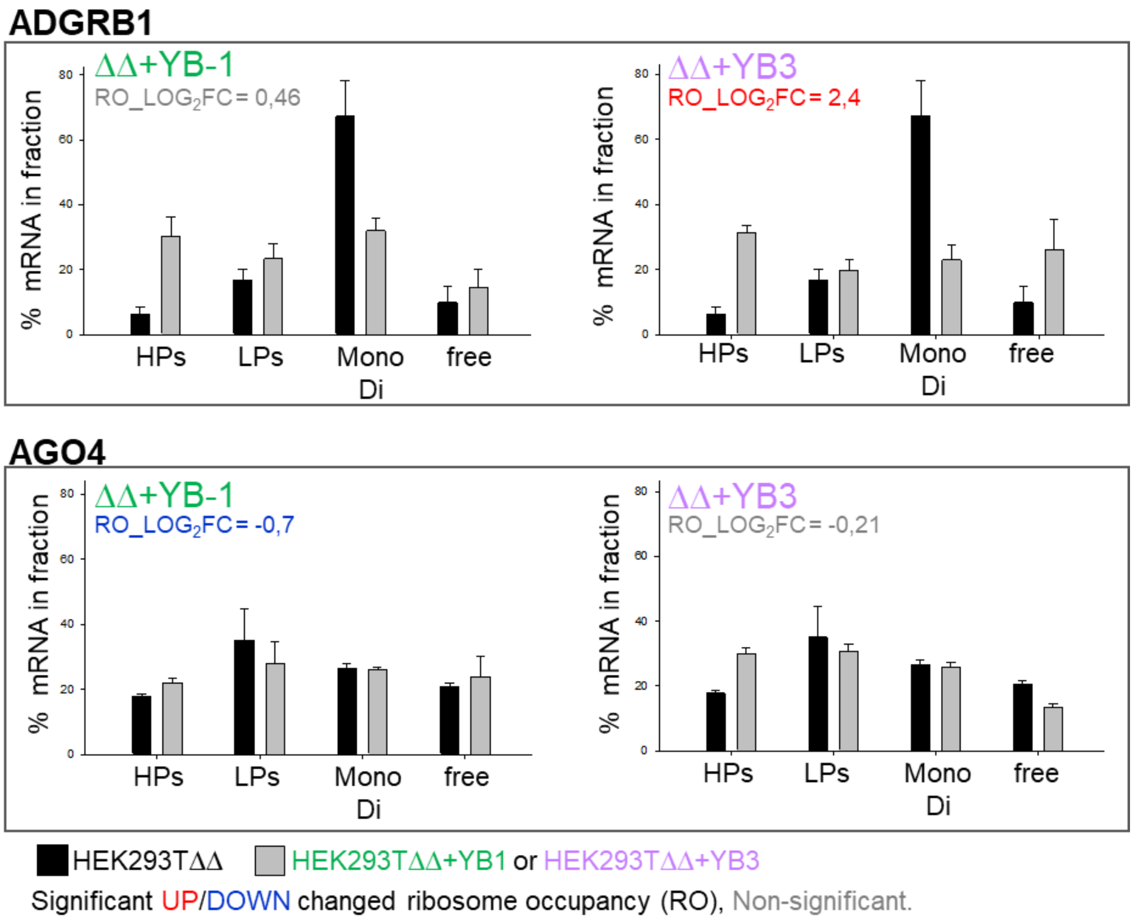

Figure S5. Verification of Ribo-Seq data by analyzing the distribution of mRNA between polysome fractions and free mRNPs. In each graph, the mRNA distribution for cell lines HEK293TΔΔ+YB-1 or HEK293TΔΔ+YB-3 (gray histogram) is compared to the HEK293TΔΔ line (black histogram). The gradient fractions are shown in Figure 1b. The fractions were collected and combined into larger ones: HPs – heavy polysomes (fractions 1-6), LPs – light polysomes (fractions 7-9), MonoDi – monosomes and disomes (fractions 10-12), free – free mRNPs (fractions 13-16). 0.1 ng of *in vitro* transcribed *Nanoluc luciferase (Nluc)* mRNA was added to each combined fraction for normalization. The mRNA abundance was measured by qRT-PCR (see Supplementary Methods 1.2). The Ribosome occupancy log2 Fold Changes obtained from high-throughput experiments are shown: significant (FDR < 0.05) UP and DOWN regulated genes are red and blue, non-significant are grey.

Figure S5 (continued)

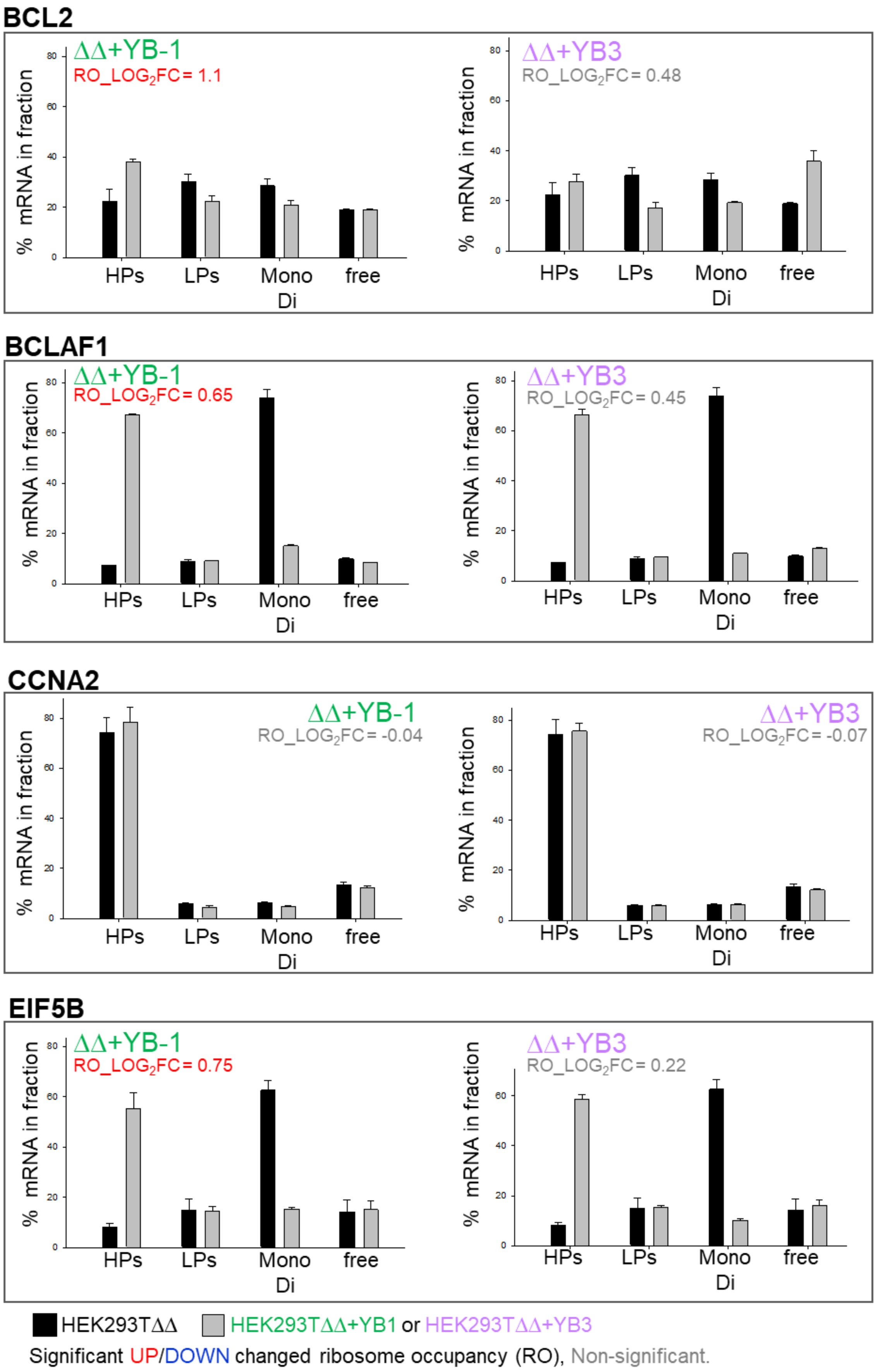

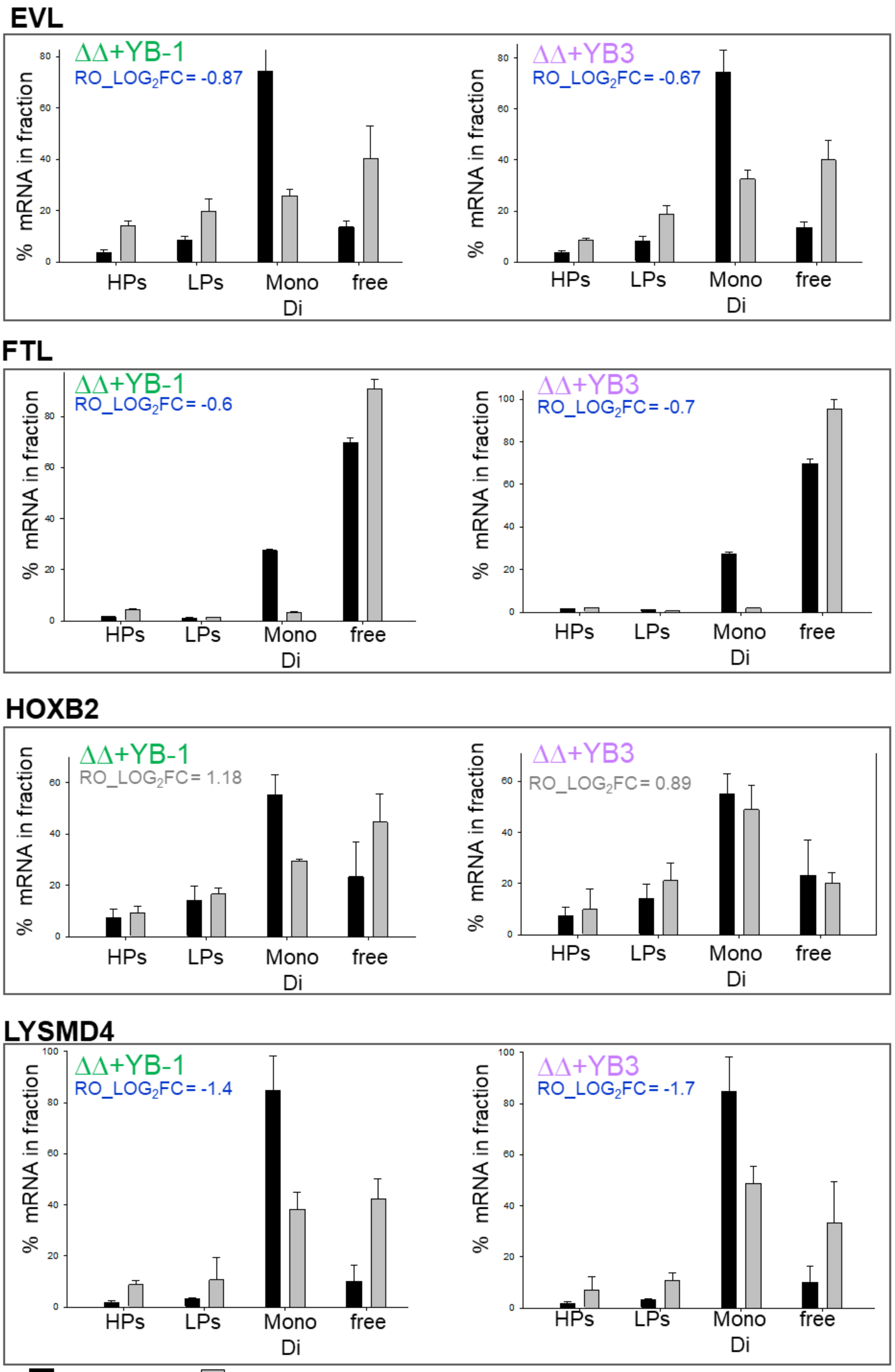

HEK293TΔΔ HEK293TΔΔ+YB1 or HEK293TΔΔ+YB3

Significant UP/DOWN changed ribosome occupancy (RO), Non-significant.

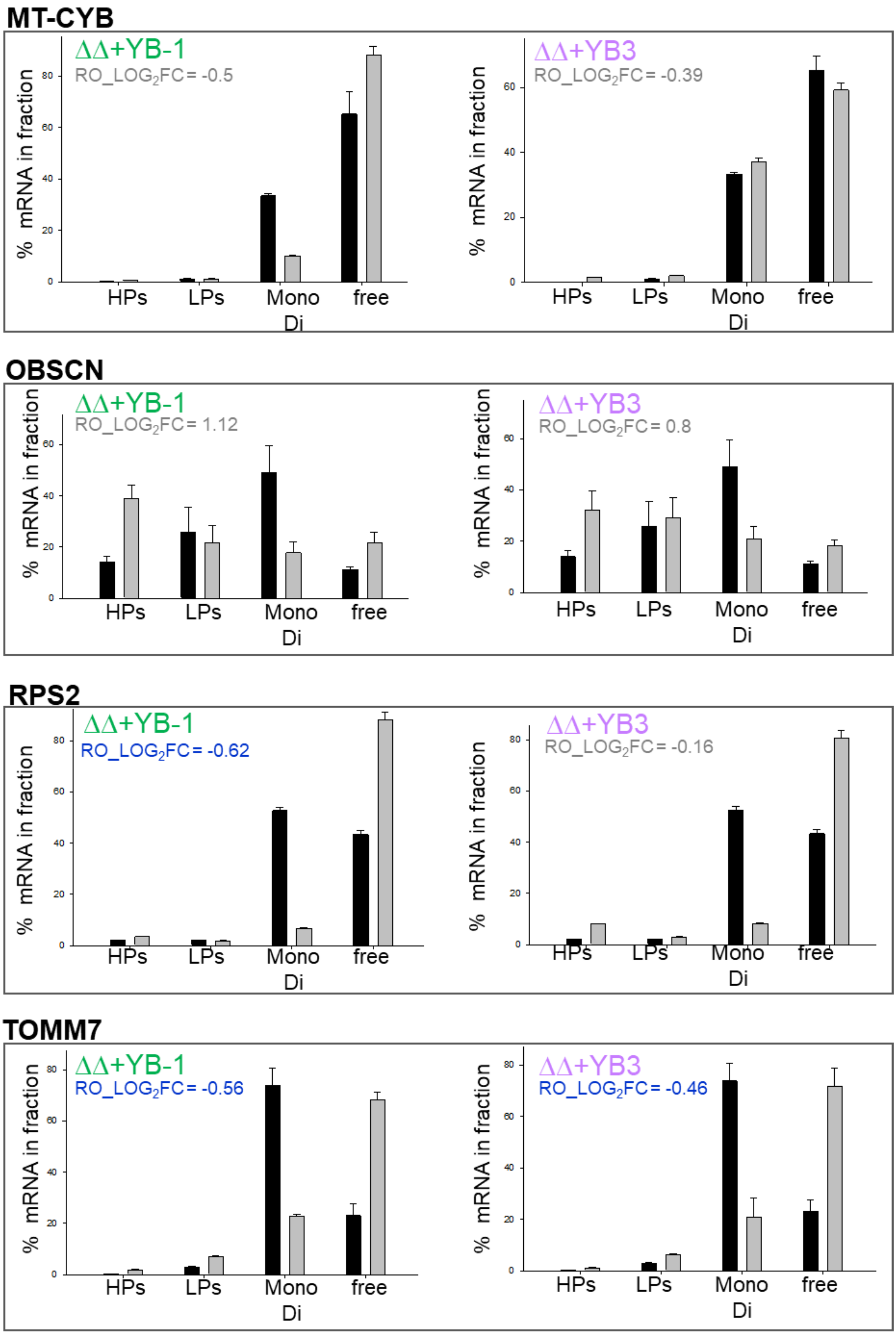

HEK293TΔΔ HEK293TΔΔ+YB1 or HEK293TΔΔ+YB3

Significant UP/DOWN changed ribosome occupancy (RO), Non-significant.

Gene (mRNA)	mRNA length (coding region length)	YB-1 vs ΔΔ	YB-3 vs ΔΔ
ADGRB1	6020 (4755)	+?	+
AGO4	7272 (2586)	-	+
BCL2	1595 (618)	+	+
BCLAF1	7491 (2763)	+	+
CCNA2	2748 (1299)	+	+
EIF5B	5741(3663)	+?	+?
EVL	1834 (1257)	?	?
FTL	871 (528)	+	+
HOXB2	1682 (1071)	-	-
LYSMD4	2770 (891)	?	?
MT-CYB	1141	+	+
OBSCN	20518 (19863)	+?	+?
RPS2	945 (882)	+	-
TOMM7	434 (168)	+	+

Table S4. Summary of Ribo-Seq data verification. Results of comparison of data from the analysis of mRNA distribution in sucrose density gradient with RiboSeq data.

«+» – changes in RiboSeq that correspond to data from the analysis of mRNA distribution in sucrose density gradient and are statistically significant (***15 genes***)

«+?» – changes in RiboSeq that correspond to data from the analysis of mRNA distribution in sucrose density gradient but are not statistically significant (***5 genes***)

«–» – changes in RiboSeq that do not correspond to the analysis of mRNA distribution in sucrose density gradient (***3 genes***)

«?» – difficulties in interpreting that sucrose gradient data (***4 genes***)
